# Synthesis of a Mixed Bis(imino)acenaphthene (BIAN) Ligand Using Aluminum Transfer Reagents

**DOI:** 10.3390/molecules31122185

**Published:** 2026-06-22

**Authors:** Kalyan V. Vasudevan, Nicholas J. Hill, Daniel K. Unruh, Andrew K. King, Alan H. Cowley, Michael Findlater

**Affiliations:** 1 Department of Chemistry, University of Texas at Austin, Austin, TX 78712, USA; 2 Department of Chemistry, University of Wisconsin Madison, Madison, WI 53706, USA; 3 Department of Chemistry, Texas Tech University, Lubbock, TX 79409, USA; 4 Department of Chemistry & Biochemistry, University of California Merced, Merced, CA 95343, USA; kingak@hiram.edu

**Keywords:** BIAN, aluminum, main group, ligand synthesis

## Abstract

Aminoalane and iminoalane transfer reagents are employed in the synthesis of ketoimines based upon an acenaphthene scaffold. One of the ketoimine species is subsequently used as a precursor for the synthesis of a mixed-BIAN ligand which features both perfluorophenyl- and *tert*-butyl substituents.

## 1. Introduction

**Figure 1:**
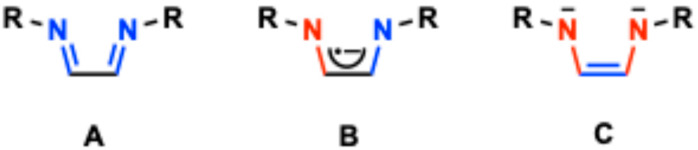
Redox-states available to a-diimine ligands in coordination complexes: (**A**) neutral ligand structure; (**B**) singly-reduced, radical anionic, ligand; (**C**) doubly-reduced, dianionic, ligand.

**Scheme 1:**
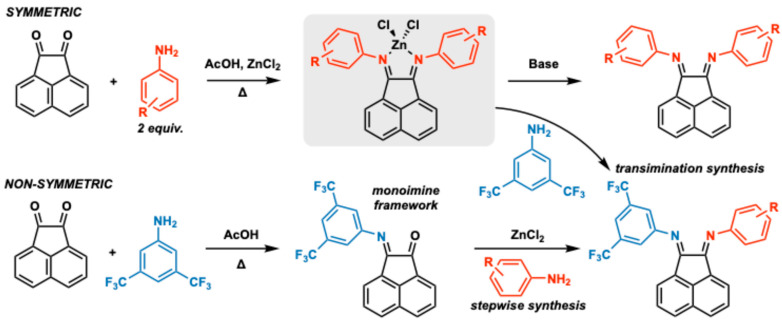
Synthetic strategies to yield symmetric Ar-BIAN compounds (**top**). Synthesis of non-symmetric Ar^1^,Ar^2^-BIAN complexes via transimination from a symmetric Ar-BIAN-Zn complexes or stepwise ZnCl_2_ condensation toward a monoimine framework (**below**) [5].

Redox non-innocent ligands and their incorporation in the coordination shell of transition metal complexes is of significant interest due to their many applications, fundamentally in catalysis [1,2,3]. This acute interest has primarily focused on the ability to manipulate the electronic structure of coordination complexes formed with such ligands and, additionally, the assessment of their subsequent catalytic behaviour [4,5,6]. The a-diimine class of redox non-innocent ligands have received widespread attention, and the ligands may occupy three distinct electronic configurations when complexed to transition metal elements (Figure 1). The ligand can adopt neutral (**A**), radical anionic (**B**) and dianionic forms (**C**) [7]. In particular, the robust framework of the bis(arylimino)acenaphthene (BIAN) ligand has emerged as a privileged ligand and has found widespread use in both coordination chemistry and catalysis (see Scheme 1 for the typical structure of a BIAN ligand) [8,9,10]. The fusion of a naphthalene ring with the a-diimine fragment both extends the conjugation of the ligand and provides a structural rigidity that is considered advantageous for metal complex stability [8].

A major advantage to the use of BIAN ligands is their ease of synthesis, which allows access to new transition metal complexes and therefore exploration of their potential applications in catalysis [4,5,6]. The preparation of a symmetrical Ar-BIAN is relatively straightforward and typically furnishes good to excellent synthetic yields [11]. In contrast, non-symmetrical ligands are usually obtained in (overall) lower isolated yields [12]. In principle, most Ar-BIAN ligand structures are accessed in a routine synthetic route via condensation reactions between the commercially available acenaphthenequinone (diketone) (ANQ) and the desired aniline under acidic conditions. For example, the commonly employed ^Dipp^BIAN (Dipp = 2,6-diisopropylphenyl) ligand can be prepared easily by heating ANQ with 2,6-diisopropylaniline for 1 h in acetic acid at reflux temperature [11]. The ability to quickly and easily diversify catalyst structure—and therefore potentially catalytic behaviour—via a robust and modular synthetic protocol is also a hallmark of BIAN ligand synthesis, although modification of reaction conditions is often required to optimize reaction yield. For example, anilines which contain strongly electron-withdrawing groups (EWG), such as trifluoromethyl groups (CF_3_, Scheme 1), typically require stronger acids and longer reaction times to obtain synthetically useful yields of such ligands [13].

Alternatively, Ragaini and coworkers deployed an interesting ‘templating’ strategy which afforded BIAN ligands bearing EWGs in high synthetic yields [14,15]. In this approach, the formation of the ligand is assisted by use of a metal templating agent, which promotes the formation of the EWG-substituted BIAN-Zn(II) or BIAN-Ni(II) complexes. As a result of the sparing solubility of the complexes in acetic acid, they can be easily isolated via simple filtration techniques. The free BIAN ligand can be isolated by treatment of the metalated complexes using common bases such as potassium carbonate (K_2_CO_3_) or sodium oxalate (Na_2_C_2_O_4_), which act to ‘de-metalate’ the complex. Using this approach, several ^Ar^BIAN ligands with electronically diverse (EWGs or electron donating groups (EDG)) substituents were prepared [11]. Through the use of these newly developed synthetic procedures, it is possible to minimize the inherent disparity in the kinetics of the condensation reactions between the two different anilines with the ANQ diketone to deliver non-symmetric BIAN ligands. Consequently, the preparation, isolation and study of non-symmetric BIAN ligand analogues have been reported upon extensively by Ragaini and coworkers [15,16].

To access non-symmetric BIAN ligands, two strategies were described [15]. The first exploits a transimination reaction which employs a symmetric BIAN-Zn complex bearing electron-withdrawing CF_3_ substituents on the *N*-arylimino moiety. A two-step process which involves condensation of ANQ with 3,5-bis(trifluoromethyl)aniline to access a monosubstituted (monoamine) structure, with subsequent condensation with an electron-rich aniline in the presence of ZnCl_2_, is often used as an alternative approach to non-symmetrical BIANs. Additionally, more complex BIAN ligands bearing extra functionalities such as O, P, and S donor atoms were also synthesized through the second stepwise strategy [17,18]. Finally, mechanochemistry (solid-state synthesis) has also been disclosed as a practical methodology to prepare indium (III) BIAN complexes via ball-milling a mixture of ANQ, an appropriate aniline, and InCl_3_ in stainless-steel grinder jars with high yields [19].

**Scheme 2:**
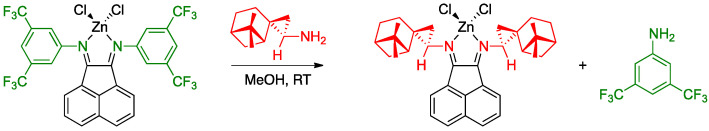
Transimination strategy for the synthesis of a chiral BIAN ligand [22].

In contrast, transimination reactions (Scheme 2) [20] are typically deployed when the direct ketone/aniline condensation is difficult. Transimination is a process most readily accomplished via substitution of electron-poor aryl groups in complexes of type [(Ar-BIAN)ZnCl_2_]. The labile nature of electron-poor substituents on the BIAN ligand prevents straightforward synthesis of mixed-BIAN ligands in which only one substituent is electron-poor. Thus, the preparation of Ar-BIAN ligands which incorporate fluorinated substituents are relatively rare [20,21] and are typically only employed to facilitate transimination reactions.

Herein, we describe the synthesis of ketoimine ligands based on the acenaphthene scaffold and the use of one such ligand for the synthesis of a mixed-BIAN ligand featuring both perfluorophenyl and *tert*-butyl substituents.

## 2. Results and Discussion

### 2.1. Synthesis and Structures

**Figure 2:**
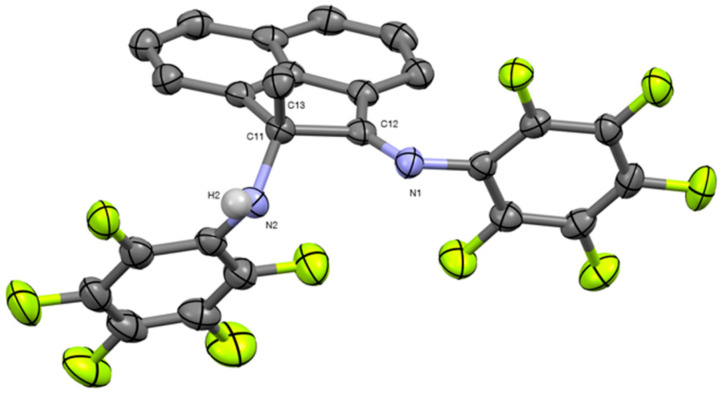
ORTEP depiction of **1** with thermal ellipsoids at 50% probability level. All hydrogen atoms except H2 have been omitted for clarity.

Our initial attempt at preparing the C_6_F_5_-BIAN ligand involved the reaction of [Me_2_Al-μ-N(H)C_6_F_5_]_2_ [23,24] and ANQ (Equation (1)). Workup of the reaction mixture and recrystallization of the resulting solid produced a crop of yellow crystals. Multinuclear NMR spectroscopy and single crystal X-ray diffraction experiments revealed the identity of the compound to be [1-methyl-2-pentafluorophenylimino-acenaphthen-1-yl)-pentafluorophenylamine] (**1**) (Figure 2). Contrary to the anticipated C_6_F_5_-BIAN ligand, the crystal structure clearly indicates the presence of a methyl group covalently attached to C(11) of the five-membered ring.
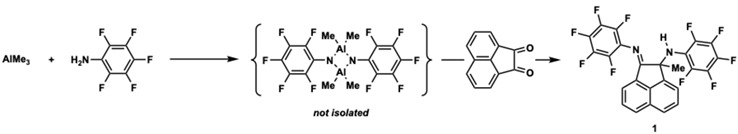
(1)

Several of the metrical parameters provide insight into the molecular structure of **1**. Thus, the C(11)-C(12) and C(12)-N(1) bond distances 1.566(3) and 1.277(3) Å are indicative of C-C single and C=N double bonds, respectively. However, the C(11)-N(2) and C(11)-C(13) bond distances of 1.474(3) and 1.523(3) Å, respectively, are both consistent with single C-N and C-C bonds. The presence of H_2_ further confirms the saturated nature of the bonds. Solution NMR data are consistent with the solid-state structure of **1**.

By analogy to the work reported by Ragaini et al. [22], the presence of this methyl group is likely a consequence of the excessive ring strain that exists in the transiently formed C_6_F_5_-BIAN ligand. It is worth noting that the incorporation of alkyl- (and other) substituents into the BIAN framework in a deliberate manner is desirable as it affords chiral BIANs [21]. Thus, the formation of **1** suggests that an aluminum transfer reagent is suitable for the synthesis of C_6_F_5_-BIAN. The presence of a reactive methyl group on the alane reagent, however, prevents isolation of the target ligand.

**Figure 3:**
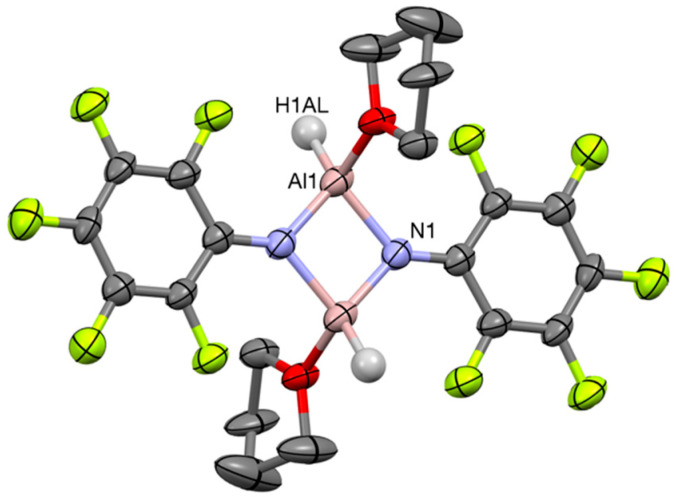
ORTEP depiction of **2a** with thermal ellipsoids at 50% probability level. All THF hydrogen atoms have been removed from clarity.

To avoid decomposition of C_6_F_5_-BIAN to **1**, it seemed reasonable to employ an alternate aluminum transfer reagent lacking the ability to methylate. Thus, the iminoalane dimer [C_6_F_5_N-Al(H)(THF)]_2_ (**2a**) was prepared by reaction of equimolar quantities of (CH_3_)_3_N-AlH_3_ and 2,3,4,5,6-pentafluoroaniline (Equation (2)). Recrystallization of the solid from THF solution at −15 °C furnished a crop of colourless crystals. The molecular structure was established based on single crystal X-ray diffraction experiments and is provided in Figure 3.
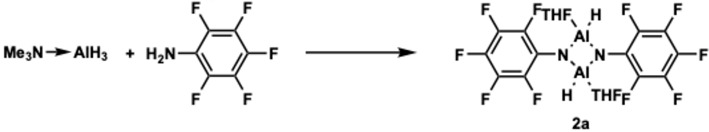
(2)

The reaction of excess **2a** with ANQ resulted in the formation of an orange solid (Equation (3)). Analysis of a solution of the crude solid via GC-MS indicated the presence of C_6_F_5_-NH_2_ in the mixture. Slow evaporation of a saturated toluene solution of **3** over 4 weeks resulted in the formation of a crop of large orange blocks. Contrary to the double-substitution observed for compound **1**, the use of [C_6_F_5_N-Al(H)(THF)]_2_ resulted in the formation of 2-pentafluorophenylimino-2H-acenaphthylen-1-one (**3**), possessing a single C_6_F_5_ unit as revealed by ^1^H-NMR and HRMS data. Variation in reaction stoichiometry and conditions always led to the isolation of ketoimine **3**, with no detectable formation of the target C_6_F_5_-BIAN ligand.
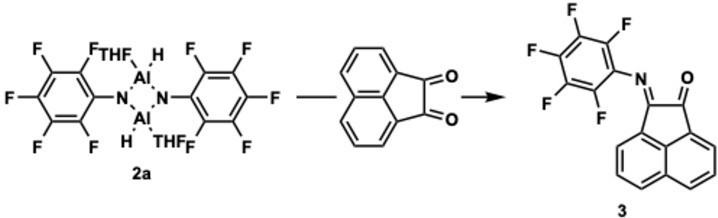
(3)

To explore the potential synthesis of BIAN ligands possessing other fluorinated substituents, we turned our attention to the 2,3,5,6-tetrafluorophenyl substituent. Thus, the combination of equimolar quantities of (CH_3_)_3_N-AlH_3_ and 2,3,5,6-tetrafluoroaniline produced a colourless powder that was subsequently treated with ANQ (Equation (4)). Workup of the reaction mixture followed by recrystallization of the crude solid resulted in a crop of orange crystals of [2-(2,3,5,6-tetrafluorophenylimino)-2H-acenaphthylen-1-one] (**4**).
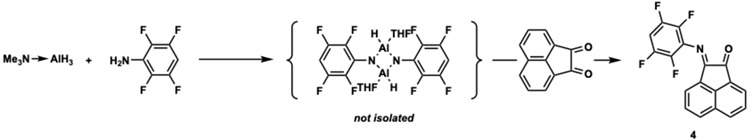
(4)

**Figure 4:**
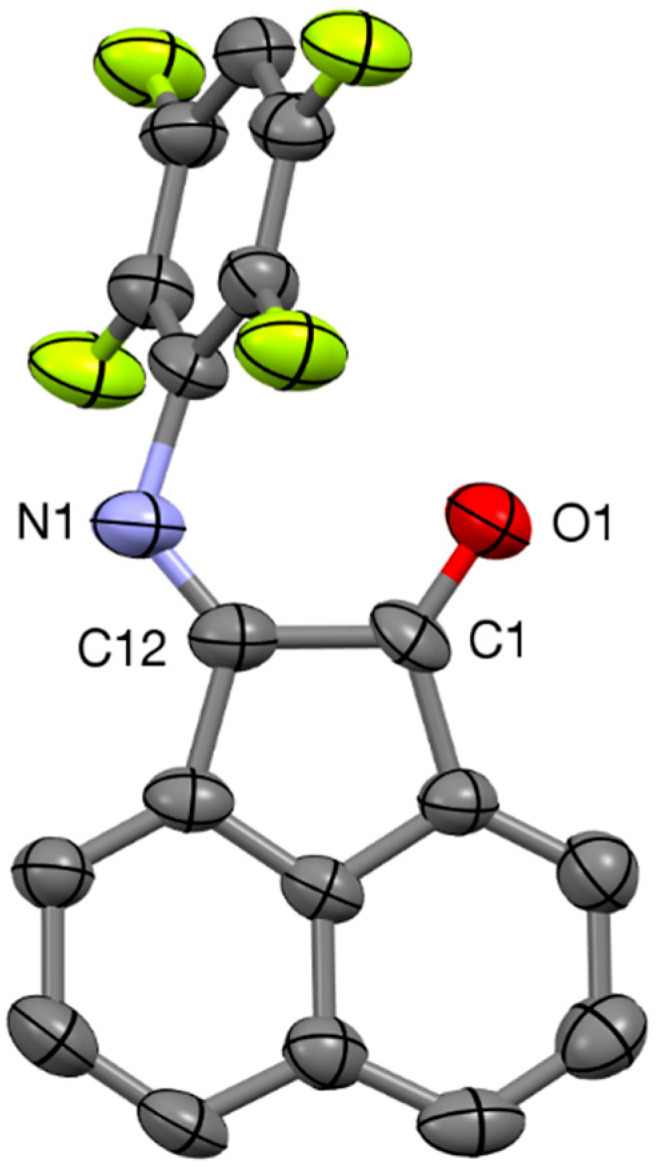
ORTEP depiction of **4** with thermal ellipsoids at 50% probability level. All hydrogen atoms and a solvent molecule (MeCN) are omitted for clarity.

In a similar fashion to the synthesis of **3**, ketoimine **4** is the exclusive product of the reaction despite the use of a large excess of aminoalane transfer reagent. The molecular structure of **4** is shown in Figure 4. The C(1)-C(12) bond distance of 1.543(14) Å and C(12)-N(1) bond length of 1.275(11) Å suggest bond orders of one and two, respectively. Similarly, the C(1)-O(1) bond distance of 1.215(11) Å clearly supports the assignment of a keto-imine ligand framework.

**Figure 5:**
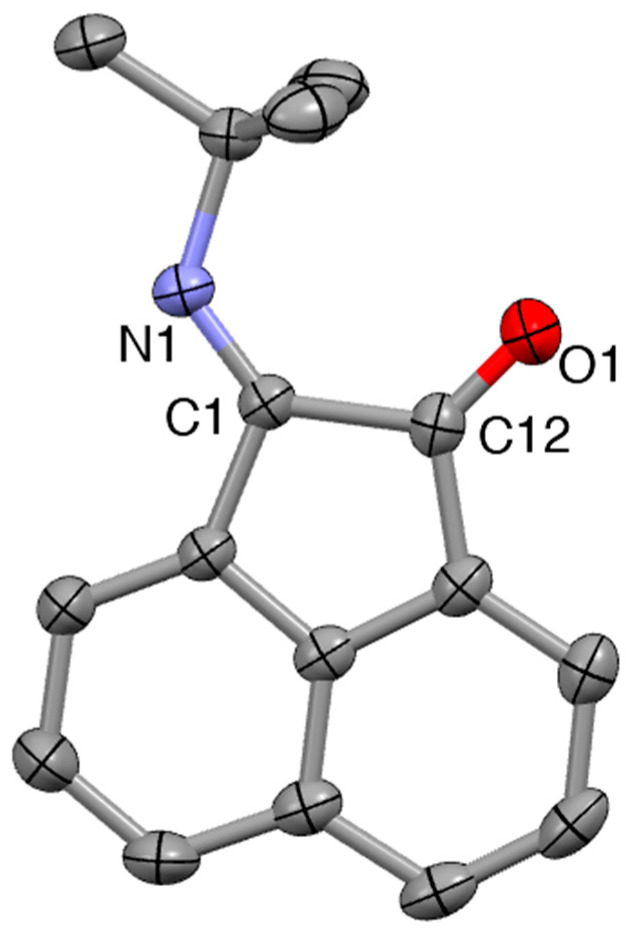
ORTEP depiction of **5** with thermal ellipsoids at 50% probability level. All hydrogen atoms are omitted for clarity.

In a similar fashion, the selective preparation of alkyl substituted BIAN ligands is also of interest [21,22]. The success in deploying the aminoalane transfer reagent (**2a**) prompted us to explore the analogous use of an alkyl-substituted derivative, [HAl(N-*^t^*Bu)]_4_ (**2b**) [23,24] in the preparation of the alkyl-substituted BIAN ligand. To our surprise, even in the presence of an excess of **2b**, reaction between ANQ and **2b** only afforded sparingly low yields of the ketoimine product, **5** (Equation (5)). Owing to the low isolated yields of **5**, characterization was achieved using mass spectrometry and ^1^H NMR spectroscopy. Fortunately, we were able to isolate single crystals of sufficient quality to allow determination of a solid-state structure, confirming the identity of a monoamine product (**5**, Figure 5).
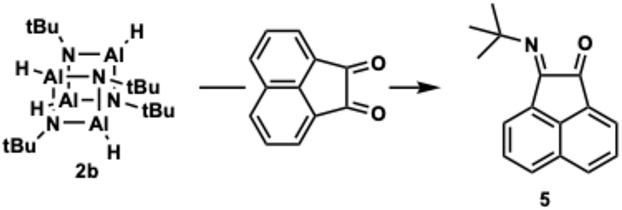
(5)

**Figure 6:**
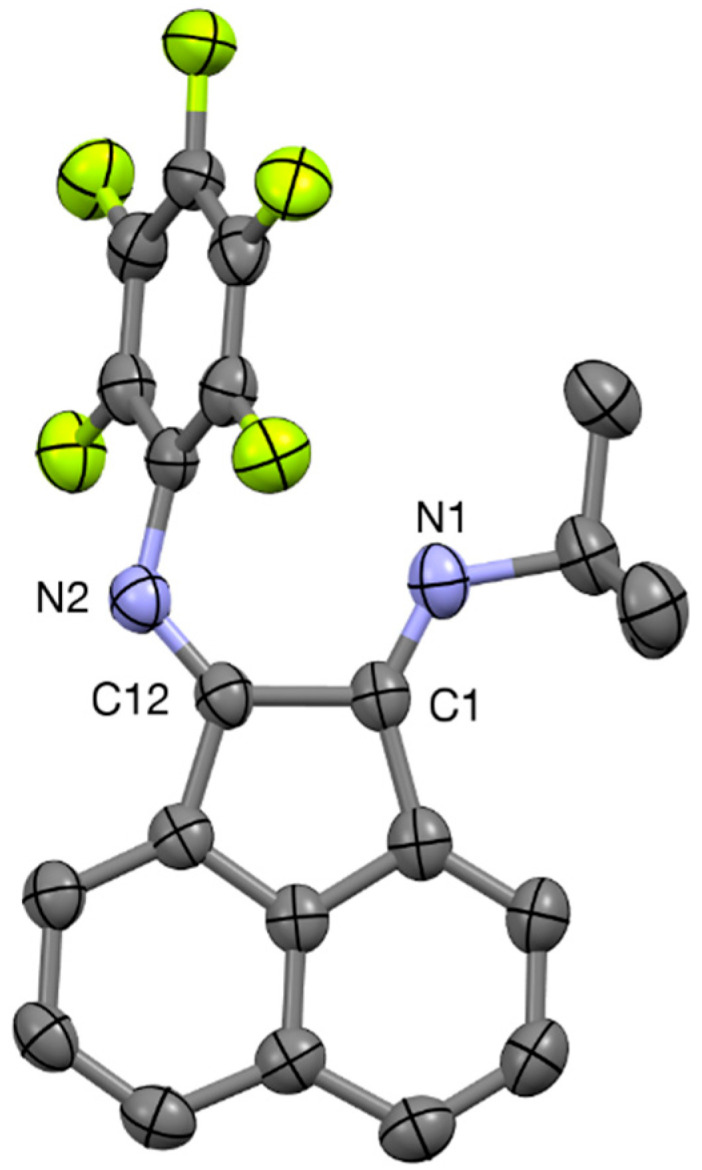
ORTEP depiction of **6** with thermal ellipsoids at 50% probability level; all hydrogen atoms omitted for clarity.

Presented with the difficulty in isolating the desired fluorinated BIAN ligands, we turned our attention to the synthesis of a mixed-BIAN ligand possessing both fluorinated and alkyl substituents. Although alkyl-BIANs have proven inaccessible via the typical condensation route employed for the aryl analogues, we speculated that the [HAl(N-*^t^*Bu)]_4_ (**2b**) [23,24] aminoalane transfer reagent used for the synthesis of the *^t^*Bu-BIAN ligand [20] could furnish a mixed-BIAN ligand via reaction with a ketoimine. Accordingly, reaction of **3** with excess **2b** followed by work-up and recrystallization resulted in a crop of yellow crystals (Equation (6)). The structure of the product (**6**) was established by NMR spectroscopy and X-ray crystallography (Figure 6).
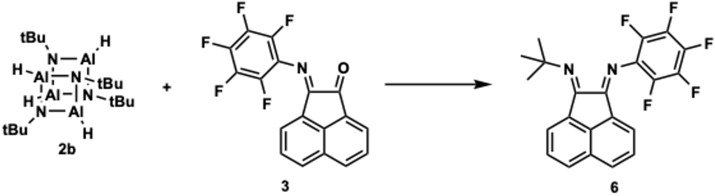
(6)

The metrical parameters of **6** unambiguously establish the successful formation of the anticipated mixed aryl-alkyl-BIAN species. Thus, the C(1)-C(12) bond distance of 1.541(4) Å falls in the expected range for a C-C single bond, and the C(1)-N(1) and C(12)-N(2) bond distances of 1.281(3) and 1.270(3) Å, respectively, are typical of a C=N double bond. Interestingly, compound **5** adopts the (*E*,*Z*) conformation in the solid state which is the conformation observed for alkyl-BIAN ligands and contrary to the vast majority of aryl-BIAN ligands that exist in the (*E*,*E*) conformation [15,16,22].

### 2.2. DFT Calculations

The ability to manipulate the redox behaviour of ligands lies at the heart of the application of redox non-innocent ligands in chemistry [1,2]. Thus, providing ready synthetic access to ligands in which the redox properties (e.g., HOMO-LUMO gap) can be controlled is of paramount importance [2]. Our naïve assumption was that a mixed BIAN ligand, in which the substituents are electronically distinct, might exhibit properties intermediate to the corresponding symmetrical analogues. To probe the electronic structure of our ligand, we performed DFT calculations on mixed BIAN **6** and symmetrical BIANs **7** (*bis*-^*t*^Bu) [21] and **8** (*bis*-C_6_F_5_) using the approach recently reported by Zysman-Colman and Matsumi [25,26]. Thus, **6**–**8** were examined in both the (*Z*,*E*) and (*E*,*E*) conformations of the imines at the B3LYP/6-31 G (2df) level of theory. In each ligand, the (*E*,*Z*) conformer is lowest in energy. The energy difference between the (*Z*,*E*) and (*E*,*E*) species is ~2.5–4.5 kcal/mol (see Supplementary Data). All the calculated species are local minima on the potential energy surface, and frontier orbital energies were obtained via an NBO calculation on the optimized structure. Given that metal chelation is facile and occurs (by definition) strictly in the (*E*,*E*) geometry, we carried out a relaxed scan of the change in C-N-C angle and subsequently took the highest energy point and performed a transition state optimization. These calculations resulted in isomerization barriers on the order of ~16–17 kcal/mol (see Supplementary Data). Although a somewhat higher value than anticipated was calculated for the barrier to isomerization, the chelate effect strongly favours metal binding, and it is plausible that metal coordination occurs in a multi-step fashion initially at the (*E*,*Z*) geometry prior to isomerization and chelation in an (*E*,*E*) geometry.

**Figure 7:**
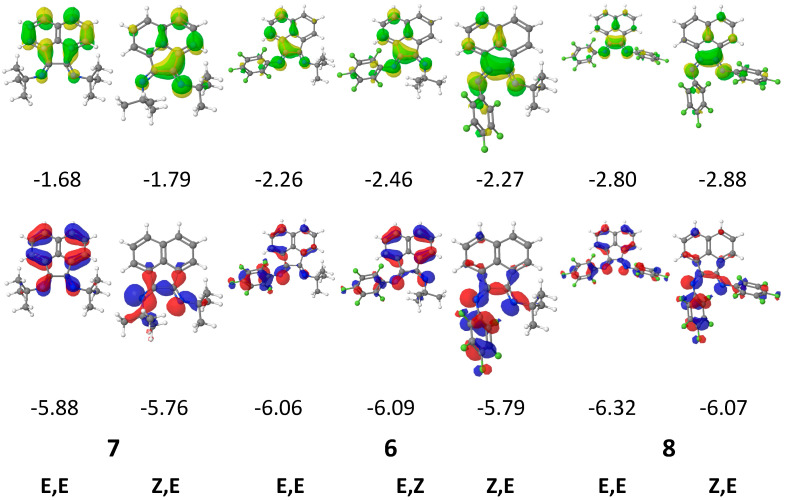
Electron density distributions of the HOMO and LUMO for **6**–**8** in the (*Z*,*E)* and (*E*,*E)* (and (*E*,*Z)* in the case of **6**) configurations at the B3LYP/6-31 G (2df) level of theory. DFT energies reported in eV.

Electron distribution images and orbital energies of **6**–**8** are shown in Figure 7. The HOMO-LUMO gap in **6** (3.63 eV) is approximately intermediate between the two homo bis-species (3.97 and 3.20 eV for **7** and **8**, respectively).

## 3. Experimental

### 3.1. Materials and Methods

All syntheses and manipulations were carried out under argon using standard Schlenk techniques or inside an inert atmosphere glovebox. Aluminum hydride, **2b**, was prepared according to published procedures [27,28]. Reaction solvents were distilled from sodium prior to use. All reagents, including organic solvents for work-up procedures, were purchased from commercial vendors and used without further purification. Low-resolution mass spectra were collected on a Finnigan MATTSQ-700 mass spectrometer (Hazlet, NJ, USA), and high-resolution mass spectra were obtained on a VG Analytical ZAB-VE sector instrument. NMR spectra were recorded at ambient temperature on a Varian Inova spectrometer (Las Vegas, NV, USA) in CDCl_3_ solution unless otherwise noted. ^1^H NMR spectra are referenced to the deuterated solvent, and ^19^F NMR chemical shifts are reported relative to Freon-11.

### 3.2. Preparation of [1-Methyl-2-pentafluorophenylimino-acenaphthen-1-yl)-pentafluorophenylamine] *(**1**)*

A solution of AlMe_3_ (30 mL, 2 M in toluene, 0.06 mol) was added dropwise to a solution of 2,3,4,5,6-pentafluoroaniline (10.98 g, 0.06 mol) in toluene (50 mL). Upon addition of the alane, the formation of a colourless precipitate was observed immediately. The reaction mixture was stirred at room temperature for a further 24 h; subsequently, the volume of the reaction mixture was decreased to ~50 mL. A suspension of ANQ (1.82 g, 100 mmol) in toluene (75 mL) was added to this mixture. The total volume was reduced to ~75 mL, and the resulting brown mixture was stirred at room temperature for 4 days. After this period, the mixture was exposed to air, and the careful addition of MeOH (dropwise) was continued until gas evolution ceased. Deionized water (75 mL) and diethyl ether (125 mL) were then added, and the mixture transferred to a separatory funnel. The organic phase was isolated, and the aqueous phase was extracted with diethyl ether (3 × 25 mL). The combined organic fractions were then washed with deionized water, dried over MgSO_4_, and filtered. The solvents were removed in vacuo to afford a viscous, brown oil which was subsequently dissolved in a minimal amount of diethyl ether, filtered, and stored at −15 °C for 4 days, affording a crop of light brown crystals of 1. Yield: 0.98 g (1.9 mmol, 19%). MS (CI^+^, CH_4_): *m*/*z* 529 [M+H]^+^; ^1^H NMR (CDCl_3_): δ 1.93 (s, 3H, CH_3_), 4.50 (s, 1H, N-H), 7.06 (d, 1H, NapC-H), 7.50 (b, 2H, NapC-H), 7.63 (t, 1H, NapC-H), 7.82 (d, 1H, NapC-H), 8.00 (d, 1H, NapC-H); ^19^F NMR (CDCl_3_): δ −152.5 (d, 1F, Ar-F), −153.2 (d, 1F, Ar-F), −154.0 (d of d, 2F, Ar-F), −162.5 (t, 1F, Ar-F), −162.8 (t, 2F, Ar-F), −164.6 (t, 2F, Ar-F), −168.2 (t, 1F, Ar-F); mp: 135 °C.

### 3.3. Preparation of [C_6_F_5_N-Al(H)(THF)]_2_ *(**2a**)*

In a dropwise fashion, a solution of 2,3,4,5,6-pentafluoroaniline (6.17 g, 33.70 mmol) in diethyl ether (50 mL) was added to a stirred solution of (CH_3_)_3_N-AlH_3_ (3.00 g, 34.70 mmol) in diethyl ether (50 mL) at room temperature. Gas evolution occurred immediately upon addition. Diethyl ether (25 mL) was added and the solution stirred under argon for 1 h, during which time the formation of a white precipitate was observed. The sealed flask was stirred at room temperature for 2 days, following at which time the solution volume was reduced to approximately 75 mL and the vessel placed in a dry ice/iPrOH bath for 20 min. The cold reaction mixture was then filtered through a glass Schlenk frit, and the white precipitate washed with cold diethyl ether (50 mL). Yield: 8.30 g (11.8 mmol, 87%). Final purification was carried out by allowing a saturated THF solution of the white solid to stand for one week at −15 °C, resulting in near quantitative formation of a crop of colourless crystalline blocks of **2a**.

### 3.4. Preparation of [2-Pentafluorophenylimino-2H-acenaphthylen-1-one] *(**3**)*

A suspension of **2** (2.0 g, 3.56 mmol) in THF (100 mL) was added to a stirred suspension of ANQ (0.43 g, 2.36 mmol). The brown-orange reaction mixture was stirred for 24 h at room temperature. The resultant mixture was then cooled to 0 °C, and isopropanol (15 mL) was added dropwise over the course of 1 min. Deionized water (350 mL) and diethyl ether (200 mL) were then added sequentially and the solution transferred to a separatory funnel. The organic phase was isolated and the aqueous phase extracted with diethyl ether (3 × 25 mL). The combined organic fractions were washed with deionized water, dried with MgSO_4_, and filtered. All solvents were removed in vacuo affording a viscous, orange oil. The crude oil was dissolved in toluene (200 mL) and was allowed to slowly evaporate over 1 month. During this time, a crop of large orange crystals was obtained. Yield: 0.21 g (0.6 mmol, 24%). MS (CI^+^, CH_4_): *m*/*z* 348 [M+H]^+^; HRMS (CI^+^, CH_4_): calcd for C_18_H_6_NF_5_O *m*/*z* 348.0448; found, 348.0451; ^1^H NMR (CDCl_3_): *δ* 6.91 (d, 1H, NapC-H), 7.01–7.20 (b, 3H, NapC-H), 7.39 (t, 1H, NapC-H), 7.90 (d, 1H, NapC-H); mp: 120 °C (dec.).

### 3.5. Preparation of [2-(2,3,5,6-Tetrafluorophenylimino)-2H-acenaphthylen-1-one] *(**4**)*

A solution of 2,3,5,6-tetrafluoroaniline (2.50 g, 15.15 mmol) in diethyl ether (15 mL) was added dropwise to a stirred solution of (CH_3_)_3_N-AlH_3_ (1.34 g, 15.05 mmol). The solution was stirred under argon for 2 h, during which time a white precipitate was formed. The mixture was filtered through a glass Schlenk frit, and the solid was washed with cold diethyl ether (25 mL). After drying, a white solid was isolated (2.30 g), dissolved in THF (75 mL), and added to a suspension of ANQ (0.20 g, 1.10 mmol) in THF (75 mL). The purple reaction mixture was heated at reflux for 2 days. The resultant mixture was then cooled to 0 °C, and iPrOH (5 mL) was added slowly over 1 min. Deionized water (75 mL) was then added to the mixture, resulting in an orange organic phase. Diethyl ether (125 mL) was added and the solution transferred to a separatory funnel. The organic phase was isolated, and the aqueous phase was extracted with diethyl ether (3 × 25 mL). The combined organic layers were washed with deionized water, dried with MgSO_4_, and filtered. The solvent was removed from the filtrate in vacuo to afford an orange solid. The orange solid was recrystallized from a saturated acetonitrile solution stored at −15 °C for 3 days. Yield: 0.10 g (0.3 mmol, 21%). MS (CI^+^, CH_4_): *m*/*z* 330 [M+H]^+^; ^1^H NMR (CDCl_3_): *δ* 7.80–7.88 (b, 2H, NapC-H), 8.00–8.13 (b, 2H, NapC-H), 8.22–8.33 (b, 2H, NapC-H), 8.61 (s, 1H, Ar-H); ^19^F NMR (CD_2_Cl_2_): *δ* −139.7 (m, 1F, Ar-F), −141.9 (m, 1F, Ar-F), −151.6 (m, 1F, Ar-F), −153.0 (m, 1F, Ar-F).

### 3.6. Preparation of 2-tert-Butylimino-2H-acenaphthylen-1-one *(**5**)*

A solution of [(HAl(N-*^t^*Bu)]_4_ (1.00 g, 2.53 mmol) in toluene (50 mL) was added to a suspension of ANQ (0.46 g, 2.53 mmol). The solution was stirred for two days at room temperature. IPA (5 mL), deionized water (50 mL) and diethyl ether (75 mL) were added sequentially, and the resulting solution was transferred to a separatory funnel. The organic phase was isolated, and the aqueous phase was back-extracted with diethyl ether (3 × 10 mL). The combined organic layers were washed with deionized water, dried with MgSO_4_ and filtered. All solvents were removed, affording a viscous, brown oil to which hexane (5 mL) was added, causing precipitation of yellow and orange solids. The precipitate was filtered and dried overnight and subsequently recrystallized from a saturated dichloromethane solution stored at −15 °C for two days. Small yellow needles were isolated in very low yield. Yield: 0.05 g (0.2 mmol, 8.3%). MS (CI^+^, CH_4_): *m*/*z* 238; ^1^H NMR (CDCl_3_): *δ* 1.47 (s, 9H, *^t^*Bu), 7.50–7.58 (b, 4H, NapC-H, 7.76 (d, 2H, NapC-H).

### 3.7. Preparation of [2-tert-Butylimino-2H-acenaphthylen-1-ylidene-pentafluorophenylamine (C_6_F_5_/^t^Bu-BIAN) *(**6**)*

A solution of [HAl(N-*^t^*Bu)]_4_ (0.60 g, 1.52 mmol) in toluene (25 mL) was added to a solution of **3** (0.175 g, 0.50 mmol). The solution was stirred at reflux for 2 days. Isopropylalcohol (5 mL), deionized water (75 mL) and diethyl ether (75 mL) were added sequentially, and the mixture was transferred to a separatory funnel. The organic phase was separated, and the aqueous phase was back-extracted with diethyl ether (3 × 10 mL). The combined organic fractions were washed with deionized water, dried over MgSO_4_, and filtered. All volatiles were removed in vacuo affording a viscous, orange-brown oil. Addition of hexanes (5 mL) resulted in the formation of yellow-orange solids. The crude solid was dissolved in a 1:1 saturated toluene/hexanes solution and stored at −15 °C for 3 days, after which time a crop of yellow crystalline needles of **6** had formed. Yield: 0.04 g (0.1 mmol, 20%). ^1^H NMR (CDCl_3_): *δ* 1.33 (s, 9H, *^t^*Bu), 7.42–7.48 (t, 1H, NapC-H), 7.51–7.61 (b, 3H, NapC-H), 7.74–7.82 (b, 2H, NapC-H); ^19^F NMR (CDCl_3_): *δ* −153.1 (d, 1F, Ar-F), −157.8 (d, 1F, Ar-F), −158.8 (t, 1F, Ar-F), −164.1 (b, 1F, Ar-F), −166.9 (t, 1F, Ar-F).

## 4. Conclusions

The synthesis of compound **6** highlights a potentially versatile synthetic methodology to unsymmetrical BIAN ligand systems. The selection of *tert*-butyl and perfluorophenyl groups is noteworthy in that BIAN species featuring these substituents are inaccessible via conventional condensation or transimination routes. Theoretical calculations support the hypothesis that mixed BIAN ligands should exhibit electronic properties intermediate to those of the parent bis-aryl and bis-alkyl species. Amino- and imino-aluminum transfer reagents could enable the synthesis of BIAN ligands possessing various combinations of sterically and electronically diverse substituents.

## Data Availability

Computational and crystallographic data available upon request—original NMR data files are unavailable due to age.

## Supplementary Materials

CCDC numbers 1473678-1473682 contain the supplementary crystallographic data for this paper. These data can be obtained free of charge from The Cambridge Crystallographic Data Centre, 12 Union Road, Cambridge CB2 1EZ, UK; fax: (+44)-1223-336-033; e-mail: deposit@ccdc.cam.ac.uk or via www.ccdc.cam.ac.uk/structures. Details of computational calculations are also supplied.
